# *MUC7* VNTR polymorphism and association with bronchial asthma in Egyptian children

**DOI:** 10.1038/s41598-022-21631-4

**Published:** 2022-11-07

**Authors:** Entsar A. Saad, Afaf M. Elsaid, Rasha M. S. Shoaib, Khaled F. Megahed, Amal N. Elsharawy

**Affiliations:** 1 Chemistry Department, Faculty of Science, Damietta University, Damietta, 34517 Egypt; 2 Genetics Unit, Children Hospital, Mansoura University, Mansoura, Egypt; 3 Food and Dairy Sciences and Technology Department, Faculty of Environmental Agricultural Sciences, Arish University, Arish, North Sinai Egypt; 4 Department of Pediatrics, Faculty of Medicine, Mansoura University, Mansoura, Egypt

**Keywords:** Biochemistry, Molecular biology

## Abstract

Overproduction of mucins in the airways donates largely to airway blockage in asthma patients. Glycoprotein MUC7 plays a role in the clearance of bacteria and has anti-candidacidal criteria. Our goal was to investigate the association between the *MUC7* variable number of tandem repeats (VNTR) polymorphism and bronchial asthma among Egyptian children. The *MUC7* VNTR polymorphism was investigated among 100 children with bronchial asthma and 100 healthy controls using polymerase chain reaction (PCR) method. Serum levels of immunoglobulin E (IgE), tumor necrosis factor-alpha (TNF-α), and transforming growth factor-beta1 (TGF-β1) were assessed by enzyme-linked immunosorbent assay (ELISA) technique. The frequencies of 6*5 genotype, 5*5 genotype, (6*5 + 5*5) genotypes, and MUC7*5 allele of the *MUC7* VNTR variant were significantly lower among asthmatic patients than controls (*p* < 0.015, OR = 0.39, 95% CI = 0.19–0.81; *p* = 0.03, OR = 0.18, 95% CI = 0.04–0.86; *p* < 0.001, OR = 0.29, 95% CI = 0.15–0.58; *p* < 0.001, OR = 0.3, 95% CI = 0.17–0.55, respectively). The (6*5 + 5*5) genotypes of the *MUC7* VNTR variant were not associated with the clinical manifestations and serum levels of IgE, TNF-α, and TGF-β1 among asthmatic patients (*p* ˃ 0.05). In conclusion, the (6*5 + 5*5) genotypes of the *MUC7* VNTR variant may have a protective role for bronchial asthma in Egyptian children.

## Introduction

Bronchial asthma is a heterogeneous disease featured by a history of different expiratory airflow limitations that may become persistent later, together with respiratory manifestations of variable intensities like cough, wheezing, breath shortness, and chest tightness^1^. It is often linked with inflammation and hyper-responsiveness of the airways conducting frequent airflow obstruction episodes due to edema and bronchospasm as well as mucus hyper-secretion^1,2^. Approximately three hundred million individuals have bronchial asthma worldwide^3^. Asthma disease has a mix of clinical manifestations and intricate pathogenesis pathways^4^. Every defined asthma phenotype has its special cluster of threatening factors^5^; host-related like age, co-infections, lifestyle, smoking, and workplace exposure, genetic like family history of asthma, and environmental like allergic sensitizers e.g. dust and smoke. At the clinical level, it is difficult to determine the severity of asthma. Pulmonary function has been extensively employed to evaluate asthma severity, though this information does not reflect the underlying asthma severity in stable clinical conditions^6^. Asthma is typically a T-helper type 2 (Th2) disease characterized by high IgE levels and eosinophilic inflammation in the airway^7^. Further, T cells, innate immune cells, and structural cells of the airways produce cytokines such as TNF-α and TGF-β1 that trigger the pathogenic features of asthma^8^.

A characteristic feature of bronchial asthma is the hyper-secretion of mucus^9^, a combination of water, ions, lipids, proteins and glycoproteins that acts as the primary line of innate defence in the respiratory tract, guarding the airways against pathogens and toxins. One of its main components are mucins, heavily glycosylated proteins with short-chain glycans bonded to the protein core. These macromolecules produced by the epithelial cells are in charge of its physicochemical characteristics. Both types of mucins (secreted and membrane-bound) serve as protecting barriers and participate in cell signalling by connecting to their receptors on immune cells^10^.

In the respiratory tract, of the twenty-one recognized mucins, eight or more mucins are expressed^11^. Human salivary mucin 7 (MUC7) protein is an antimicrobial protein with antibacterial and antifungal activities. It is a mini secreted mucin (MG2; ∼ 125 kD) expressed predominantly in the saliva and the respiratory tract^12^. It is a monomer of 377 amino acid residues^13^. It has a histatin domain with anti-microbial activity at its N-terminus and a central domain that holds 5 or 6 tandem repeats (PTS repeats)^12^. Each repeat comprises 23 amino acids that have plenty of proline, threonine, and serine, and thus possesses plenty of potential O-linked glycosylation sites^13^. The MUC7 task is not sufficiently clarified. However, it is supposed to take part in defending respiratory and oral epithelia against microbial infection via reacting with pathogens and their clearance. It can reduce surface adhesions preventing bacteria from colonization. Being a glycoprotein of saliva, it participates in chewing, talking, swallowing, and oral cavity lubrication^14^.

The *MUC7* gene extends about 10 kb on chromosome 4q13.3. It has 3-exons and 2-introns. Intron 1 locates in the *MUC7* cDNA's 5-prime untranslated area and extends about 1.7 kb, and intron 2 extends roughly 6 kb. The whole region that codes the secreted peptide is lying in exon 3 that spreads along about 2.2 kb^15^.

The *MUC7* gene harbours one variable number of tandem repeat (VNTR) polymorphism. The most common allele of this VNTR, named MUC7*6, includes 6 tandem repeats, each consisting of 69 nucleotides. There are other two alleles, one carrying five repeats (MUC7*5), and a rare allele with eight repeat units (MUC7*8). These repeats are very similar but not identical, incorporating between 1 and 7 nucleotide substitutions between them^16^.

Because of the rareness of reports regarding the *MUC7* VNTR variant and the bronchial asthma risk, we conducted the present study to evaluate the frequency of the *MUC7* VNTR variant, for the first time, among Egyptian children with bronchial asthma. We also studied the associations between this variant and serum levels of some asthma biomarkers (IgE, TNF-α, TGF-β1) and the clinical manifestations of the disease.

## Methods

### Study participants

This preliminary case–control study involved 200 individuals of Egyptian ethnicity, 100 children with bronchial asthma (57 boys and 43 girls) and 100 healthy controls (61 boys and 39 girls). All participants were enrolled in the period extended from October 2019 to December 2020. Children with asthma were recruited at the Allergy and Pulmonology Outpatient Clinics of Mansoura University Children's Hospital, Egypt, while controls were recruited at the General Outpatient Clinics of the same hospital. Diagnosis of asthma was done based on Global Initiative for Asthma guidelines^17^. Patients suffering from cystic fibrosis, congenital heart disease, immunological deficiency, or who had consumed antibiotics, hormones, or asthma medicines in the two weeks preceding enrolment were excluded. Direct interviews and patients' files were used to obtain the clinical data of the study participants.

### Ethics approval and consent to participate

The Institutional Review Board of the Faculty of Medicine, Mansoura University, Egypt approved the study (Code number: R.21.12.1555.R1). Informed written consent was acquired from legal guardians of all investigation participants with the declaration of data privacy. The study was performed in accordance with the Declaration of Helsinki.

### Clinical evaluation and skin tests

Atopic individuals were defined as those with a total serum IgE level ≥ 300 ng/mL and skin prick tests performed according to the guidelines of European Academy of Allergy and Clinical Immunology with a battery of common aeroallergens^18^.

### Sample collection and analysis

From each study participant, 5 mL of peripheral venous blood was withdrawn and divided into two tubes; 4 mL were collected without an anticoagulant and centrifuged for 15 min at 5000 rpm to obtain serum for estimating the levels of IgE, TNF-α, and TGF-β1, and 1 mL blood was placed in a test tube containing EDTA for DNA extraction.

### Estimation of serum parameters

Serum IgE, TNF-α, and TGF-β1 levels were assessed by the quantitative ELISA kits Abcam, ab178659, USA; Abcam, ab181421, USA; Invitrogen, BMS249-4, USA; respectively.

### Extraction of genomic DNA

Genomic DNA extraction was carried out from 200 µL of blood using the Generation DNA Purification capture column kit (BioFlux, China). The NanoDropTM 1000 Spectrophotometer was used to estimate DNA level and purity.

### Genotyping of the *MUC7* VNTR polymorphism by PCR method

The genotyping of the VNTR (69 bp repeat) polymorphism in exon 3 of the *MUC7* gene was achieved using the PCR procedure described by Kirkbride et al.^16^. Primers were obtained through Applied Biosystems' Assays-by-Demand SNP genotyping service (Foster City, California, United States). Oligonucleotide primers had the following sequences: sense 5'-GTAGCTACATTAGCACCAGTG-3' and antisense 5'-TTCAGAAGTGTCAGGTGCAAG-3'. Each PCR reaction comprised 10 µL 10 × PCR buffer pH 9.0, 10 µL 2 mM dNTPs, 1 µM sense and antisense primers, 1 µL (0.5 µg) DNA, 0.5 µL (2.5 U) Taq polymerase (Promega, Southampton, UK), and water to a final volume of 100 µL. Thermo-cycling steps involved 2 min of denaturation at 94 °C, then 25 cycles of denaturation at 95 °C for 30 s, an annealing step at 60 °C for 30 s, and extension at 72 °C for 30 s. A final extension was carried out at 72 °C for 6 min. PCR yields were electrophoresed on 2% agarose gel and visualized using ethidium bromide under ultraviolet illumination. The MUC7*6-allele (six repeats) was detected at 590 bp, while MUC7*5-allele (five repeats) was found at 521 bp. The MUC7*8-allele (eight repeats) was detected at 710 bp. These products were photographed by a digital camera. We selected more than 10% of the samples randomly for repeated genotyping. The results were completely consistent.

### Statistical analysis

Statistical analyses were performed using the Statistical Package for Social Science (SPSS, version 26, Thousand Oaks, CA). The statistical power was calculated using the G*power software version 3.1.9.4 (http://www.gpower.hhu.de/). This case–control study with the alpha error probability of 0.05 and the total sample size of 200 children for two groups could provide a statistical power of 92.7% with a medium effect size of 0.30. Numbers and percentages were used to represent qualitative data. The non-parametric data was processed using the Mann–Whitney test and expressed as the median and interquartile range (IQR) after testing normality using the Kolmogorov–Smirnov test. Differences between groups were evaluated using Chi square or Mann–Whitney U tests for categorical and continuous variables, respectively. Chi square test was used to assess genotype and allele frequency differences. Odds ratio (OR) and 95 percent confidence interval (CI) values were calculated. To avoid the disagreement issues raised by the difference of age and sex of the participants, we performed the Chi square crude odd ratio followed by adjusted Chi square odd ratio by sex and age, and lastly we carried out Bonferroni-adjustment. Bonferroni *p*-value was calculated as: original *p*-value * number of comparatives (5 for genotypes or 3 for alleles). The significance level was set at *p* ≤ 0.05.

## Results

### The basic characteristics of the studied groups

Demographic, clinical, and biochemical data of the studied groups are summarized in Table 1. There were no statistically significant dissimilarities seen with age and sex among the studied groups (*p* = 0.36 and *p* = 0.67, respectively). Additionally, there was no statistical significance noticed with passive smoking (*p* = 0.1). However, there were significant dissimilarities with family history, Forced Expiratory Volume (FEV1), Forced Vital Capacity (FVC), and FEV1/FVC between the studied groups (*p* < 0.001) indicating that asthma patients had lower lung function. Moreover, the levels of IgE, TNF-α, and TGF-β1 were significantly elevated among bronchial asthma patients compared to healthy controls (*p* < 0.001).

**Table 1:** Demographic, clinical and biochemical data of the studied groups.

Parameters	Asthmatic patients (n = 100)	Controls (n = 100)	*p*
**Age (years)**	10 (8–12.5)	9.5 (8–12.0)	0.36
**Sex: male/female; n**	57 / 43	61 / 39	0.67
**Positive family history**	62	0	**< 0.001**
**Positive passive smoking**	40	28	0.1
**Duration of illness (years)**	4.5 (3.3–6.5)	-	-
**FEV1 (%)**	75.2 (68.8–79.2)	103 (99–107)	**< 0.001**
**FVC (%)**	86.7 (73.9–95.4)	107.5 (100.5–110)	**< 0.001**
**FEV1/FVC**	81.6 (78.8–107.9)	98.6 (93.8–100)	**< 0.001**
**Asthma severity degree; n**			
Intermittent	2	–	–
Mild persistent	12		
Moderate persistent	48		
Severe persistent	38		
**Positive associated allergic rhinitis**	89	–	–
**Positive skin prick test**	72	–	–
**Atopic asthma/non-atopic asthma; n**	50/50	–	–
**ICS (yes/no)**	82/18	–	–
**Serum IgE level (ng/mL)**	139 (56.1–304.3)	4.0 (2–6)	** < 0.001**
**Serum TNF-α level (pg/mL)**	506 (284.5–670.5)	87 (67–102)	** < 0.001**
**Serum TGF-β1 level (pg/mL)**	476 (344–632)	77.5 (61–91)	** < 0.001**

Data are presented as numbers with percentages or median with interquartile range. Chi square and Mann–Whitney U tests were applied.

*FEV1* forced expiratory volume, *FVC* forced vital capacity, *ICS* inhaled corticosteroids, *IgE* immunoglobulin E, *TNF-α* tumor necrosis factor-alpha, *TGF-β1* transforming growth factor-beta 1, *IQR* interquartile range, *n* number of patients, *p* probability, probability value was considered significant at *p* < 0.05.

### The genotypic and allelic frequencies of the *MUC7* VNTR variant in the studied groups

Asthma patients displayed significantly fewer frequency of 6*5 genotype of the *MUC7* VNTR variant than controls (14% vs. 29%; OR = 0.25 and 95% CI = 0.16–0.75, *p* = 0.045). The frequency of (6*5 + 5*5) genotypes was significantly lower in asthmatic cases than healthy controls (16% vs. 39%; OR = 0.19 and 95% CI = 0.12–0.42, *p* = 0.002). The MUC7*5-allele frequency was significantly low among asthmatic patients compared to controls (9% vs. 24.5%, OR = 0.23, 95% CI = 0.12–0.49, *p* = 0.00015) (Table 2).

**Table 2:** Genotypes and allelic frequencies of *MUC7* VNTR in asthmatic patients and controls.

Gene polymorphisms	Asthmatic patients (n = 100) n (%)	Controls (n = 100) n (%)	Comparisons	Adjusted OR (95% CI)	*p*^a^	*p*^b^
6*6	76 (76%)	58 (58%)				
6*5	14 (14%)	29 (29%)	(6*5) vs. (6*6 + 5*5 + 6*8 + 8*8)	0.25 (0.16–0.75)	**0.009**	**0.045**
5*5	2 (2%)	10 (10%)	(5*5) vs. (6*6 + 6*5 + 6*8 + 8*8)	0.15 (0.02–0.79)	**0.02**	**0.1**
6*8	5 (5%)	2 (2%)				
8*8	3 (3%)	1 (1%)	(6*5 + 5*5) vs. (6*6 + 6*8 + 8*8)			
				0.19 (0.12–0.42)	**0.0004**	**0.002**
MUC7*5	18 (9%)	49 (24.5%)	**5 vs. 6 + 8**			
MUC7*6	171 (85.5%)	147 (73.5%)		0.23 (0.12–0.49)	**0.00005**	**0.00015**
MUC7*8	11 (5.5%)	4 (2%)				

Data are presented as numbers with percentages.

*p*^a^: *p*-value was calculated by Chi square test after adjusting for sex and age. *p*^b^: *p*-value after Bonferroni correction. Bonferroni *p*-value was calculated as: original *p*-value * number of comparatives; number of comparatives was 5 for genotypes and 3 for alleles.

*n* number, *CI* confidence interval, *OR* odds ratio; *p* ≤ 0.05 is significant.

### The association of the *MUC7* VNTR variant with bronchial asthma risk in atopic and non-atopic asthmatic patients

In respect to the atopic and non-atopic patients, the frequency of (6*5 + 5*5) genotypes was significantly decreased within atopic asthmatic patients compared to controls (10% vs. 39%; OR = 0.15 and 95% CI = 0.02–0.42, p = 0.0015). Contrarily, the frequency of (6*5 + 5*5) genotypes was insignificantly decreased in non-atopic asthmatic patients than in controls (22% vs. 39%; OR = 0.39 and 95% CI = 0.16–0.85, p = 0.15). Furthermore, the atopic asthmatic patients exhibited not significant association with the (6*5 + 5*5) genotypes of *MUC7* VNTR compared to non-atopic asthmatic patients (10% vs. 22%; OR = 0.25 and 95% CI = 0.11–1.09, p = 0.65). The MUC7*5-allele frequency was significantly lower among atopic and non-atopic asthmatic patients than controls (6% vs. 24.5%, OR = 0.16, 95% CI = 0.05–0.42, p = 0.0003; 12% vs. 24.5%, OR = 0.35, 95% CI = 0.17–0.78, p = 0.024; respectively). While the MUC7*5-allele frequency revealed no significant difference between the atopic and non-atopic asthmatic patients (6% vs. 12%; OR = 0.44 and 95% CI = 0.14–1.1, p = 0.6) (Table 3).

**Table 3:** Genotypes and allelic frequencies of *MUC7* VNTR in atopic and non-atopic asthmatic patients and controls.

Gene polymorphisms	Asthmatic patients (n = 100) n (%)		Controls (n = 100) n (%)	Comparisons		Adjusted OR (95% CI)	*p*^a^	*p*^b^
	Atopic (n = 50)	Non-atopic (n = 50)						
6*6	42 (84%)	34 (68%)	58 (58%)					
6*5	4 (8%)	10 (20%)	29 (29%)					
5*5	1 (2%)	1 (2%)	10 (10%)					
6*8	2 (4%)	3 (6%)	2 (2%)					
8*8	1 (2%)	2 (4%)	1 (1%)					
				(6*5 + 5*5) vs. (6*6 + 6*8 + 8*8)	**Atopic vs. controls**	0.15 (0.02–0.42)	**0.0003**	**0.0015**
					**Non-atopic vs. controls**	0.39 (0.16–0.85)	**0.03**	**0.15**
					**Atopic vs. non-atopic**	0.25 (0.11–1.09)	0.13	0.65
MUC7*5	6 (6%)	12 (12%)	49 (24.5%)	**5 vs. 6 + 8**	**Atopic vs. controls**	0.16 (0.05–0.42)	**0.0001**	**0.0003**
MUC7*6	90 (90%)	81 (81%)	147 (73.5%)		**Non-atopic vs. controls**	0.35 (0.17–0.78)	**0.008**	**0.024**
MUC7*8	4 (4%)	7 (7%)	4 (2%)		**Atopic vs. non-atopic**	0.44 (0.14–1.1)	0.20	0.6

Data are presented as numbers with percentages.

*p*^a^: *p*-value was calculated by Chi square test after adjusting for sex and age. *p*^b^: *p*-value after Bonferroni correction. Bonferroni *p*-value was calculated as: original *p*-value * number of comparatives; number of comparatives was 5 for genotypes and 3 for alleles.

*n* number, *CI* confidence interval, *OR* odds ratio; *p* ≤ 0.05 is significant.

### The associations of the *MUC7* VNTR variant with the demographic, clinical and biochemical parameters of all asthmatic patients

No significant associations existed between *MUC7* VNTR genotypes and any of the demographic, clinical, or biochemical data of all asthmatic patients (Table 4). The *MUC7* VNTR variant was not associated with higher levels of serum IgE, TNF-α, and TGF-β1 levels in all asthmatic patients (*p* = 0.16; *p* = 0.08; and *p* = 0.22, respectively).

**Table 4:** Associations of *MUC7* VNTR genotypes with demographic, clinical features, and biochemical measurements in asthmatic patients.

Parameters	*MUC7* VNTR		*p*
	(6*5 + 5*5) (n = 16)	(6*6 + 6*8 + 8*8) (n = 84)	
**Age (years)**	8.5 (7.1–11)	10 (8–13.3)	0.11
**Sex: male/female; n**	9/7	48/36	1.0
**Positive family history**	11	51	0.59
**Positive passive smoking**	8	32	0.41
**Duration of illness (years)**	5 (3.1–6.5)	4.5 (3.3–6.5)	0.85
**FEV1 (%)**	75 (70.2–78.3)	75.5 (67.3–79.4)	0.89
**FVC (%)**	93.4 (86.5–94.5)	83.9 (72.3–95.6)	0.24
**FEV1/FVC**	81.3 (77.9–105.6)	81.7 (78.9–107.9)	0.55
**Asthma severity degree; n (%)**			
Intermittent	0 (0%)	2 (2.4%)	0.36
Mild persistent	0 (0%)	12 (14.3%)	
Moderate persistent	10 (62.5%)	38 (45.2%)	
Severe persistent	6 (37.5%)	32 (38.1%)	
**Positive associated allergic rhinitis**	14	75	1.0
**Positive skin prick test**	11	61	0.77
**ICS (yes/no)**	14/2	68/16	0.73
**IgE level (ng/mL)**	61.5 (49.8–234.2)	161.3 (61–334.2)	0.16
**TNF-α level (pg/mL)**	339 (123.3–615.8)	538.5 (322.5–742.8)	0.08
**TGF-β1 level (pg/mL)**	432 (423.8–553.8)	499.5 (342.5–652.8)	0.47

Data are presented as numbers with percentages or median with interquartile range. Chi square and Mann–Whitney U tests were applied.

*FEV1* forced expiratory volume, *FVC* forced vital capacity, *ICS* inhaled corticosteroids, *IgE* immunoglobulin E, *TNF-α* tumor necrosis factor-alpha, *TGF-β1* transforming growth factor-beta 1, *IQR* interquartile range, *n* number of patients, *p* probability, probability value was considered significant at *p* < 0.05.

### The associations of the *MUC7* VNTR variant with the demographic, clinical and biochemical parameters of atopic and non-atopic asthmatic patients

There were no significant associations seen between *MUC7* VNTR genotypes and any of the demographic, clinical, or biochemical data of the atopic and non-atopic asthmatic patients (Table 5). The *MUC7* VNTR variant was not associated with the higher levels of serum IgE, TNF-α, and TGF-β1 in atopic and non-atopic asthmatic patients (*p* = 0.76, 0.64; *p* = 0.49, 0.13; and *p* = 0.49, 0.12, respectively).

**Table 5:** Associations of *MUC7 VNTR* genotypes with demographic, clinical features, and biochemical measurements in atopic and non-atopic asthmatic patients.

Parameters	Atopic asthmatic (n = 50)			Non-atopic asthmatic (n = 50)		
	(6*5 + 5*5) (n = 5)	(6*6 + 6*8 + 8*8) (n = 45)	*p*	(6*5 + 5*5) (n = 11)	(6*6 + 6*8 + 8*8) (n = 39)	*p*
**Age (years)**	8 (7.5–11)	10 (8–12)	0.27	8.5 (7–13.5)	11 (8.5–14.5)	0.24
**Sex: male/female; n**	3/2	30/15	1.0	6/5	18/21	0.74
**Positive family history**	4	27	0.64	7	24	1.0
**Positive passive smoking**	2	18	1.0	6	14	0.31
**Duration of illness (years)**	3.5 (2–6.8)	4.5 (3–6.4)	0.49	6 (3.5–6.5)	4.5 (3.5–7.5)	0.72
**FEV1 (%)**	82 (69.5–97.5)	76 (65–82.5)	0.25	74.9 (69.9–75.8)	74.9 (68–78.1)	0.73
**FVC (%)**	76 (68–87)	73 (66–79)	0.37	94 (92.9–96.4)	95.4 (89.7–97.9)	0.49
**FEV1/FVC**	110.8 (101.6–112.1)	106.9 (94.1–111.2)	0.31	79 (77.7–81.6)	79.3 (75.5–80.9)	0.74
**Asthma severity degree; n (%)**						
Intermittent	0 (0%)	2 (4.4%)	0.55	0 (0%)	0 (0%)	0.49
Mild persistent	0 (0%)	5 (11.1%)		0 (0%)	7 (17.9%)	
Moderate persistent	3 (60%)	21 (46.7%)		7 (63.6%)	17 (43.6%)	
Severe persistent	2 (40%)	17 (37.8%)		4 (36.4%)	15 (38.5%)	
**Positive skin prick test**	4	32	1.0	7	29	0.48
**ICS (yes/no)**	4/1	37/8	1.0	10/1	31/8	0.66
**IgE level (ng/mL)**	343.9 (89.1–364.9)	245 (143.3–365.8)	0.76	53 (49–77)	71 (38–197)	0.64
**TNF-α level (pg/mL)**	501 (262–709.5)	589 (364.5–765)	0.49	213 (116–399)	487 (199–654)	0.13
**TGF-β1 level (pg/mL)**	523 (465–649)	455 (342–653.5)	0.49	432 (332–465)	511 (422–632)	0.12

Data are presented as numbers with percentages or median with interquartile range. Chi square and Mann–Whitney U tests were applied.

*FEV1* forced expiratory volume, *FVC* forced vital capacity, *ICS* inhaled corticosteroids, *IgE* immunoglobulin E, *TNF-α* tumor necrosis factor-alpha, *TGF-β1* transforming growth factor-beta 1, *IQR* interquartile range, *n* number of patients, *p* probability, probability value was considered significant at *p* < 0.05.

## Discussion

In asthmatic patients, extra-secreted mucus and mucins in the airways share significantly in airway blockage^19^. Thus, it was presumed that there was a link between *MUC* genes and asthma^17^. As far as we know, our study is the first to investigate the genotypic and allelic frequencies of the *MUC7* VNTR variant among Egyptian children with bronchial asthma as well as to evaluate the associations between this variant and serum IgE, TNF-α, and TGF-β1 levels, and the clinical manifestation of the asthma disease.

The present study showed a significant association of the (6*5 + 5*5) genotypes and MUC7*5 allele of the *MUC7* VNTR variant with a lowered risk of bronchial asthma. These results illuminate the MUC7*5 allele as a protector in bronchial asthma. Kirkbride et al.^16^ reported similar results. They found that the MUC7*5 allele was less prevalent in the Northern European asthmatic group. Further, a later study announced that the MUC7*5 allele is associated with a lowered risk of a diagnosis of asthma in an African American population^20^.

Despite MUC7*6 and MUC7*5 were reported as the most prevailing alleles, in different cohorts, rare alleles were recognized. The rare allele MUC7*8 led to an increase in the number and structure rearrangement of the encoded tandem repeat (TR) domains. MUC7*8 allele was identified in our present study in both patients and controls (5.5% and 2%, respectively). Similar to our findings, the MUC7*8 allele was recognized in a Northern European asthmatic patient in the study of Kirkbride et al.^16^.

Links between the repeat units number of *MUC* genes' alleles and disease susceptibility have been reported in some research^16,21–25^. It was reported that *MUC7* alleles with the least tandem repeat level are the most stable at the genetic level^26–28^. It was explored that the MUC7*5 allele lacks the fourth unit within the tandem repeat region indicating a short-length tandem repeat region by 16.6% missing probable 9 O-glycosylation locations^16^. Since MUC7 is rich in O-glycosylations, significant allelic variability in mucin length is expected to affect both the number of glycosylation sites and the other physicochemical aspects^29^. Both could have functional implications and lead to differences in disease susceptibility^20^. It appears logical that the association couples variations in allelic length with bacterial interactions where binding of bacteria is speculated to be the task of the glycosylated domain, which permits bacterial clearance from the epithelial surfaces^30^.

The connexion between single nucleotide polymorphism (SNP) of genes and numerous clinical diseases has been tested and ascertained in many different surveys^31–34^. One hundred percent linkage disequilibrium was recognized in rs998210 SNP with the MUC7*5 polymorphism in a Northern European group^35^. In an African American cohort, rs998210 SNP was significantly linked, with less than 100% linkage disequilibrium, to the MUC7*5 polymorphism^20^. Due to its location within a not-known motif region, this SNP was deemed not functional^35^.

The more acceptable likely mode of action for the protecting impact of *MUC7*∗5 is via alterations in bacterial interaction and clearance. MUC7 interaction with bacteria is mediated by the MUC7 glycosylated N-terminal domain. In the case of the expressed shorter glycoprotein coded by the *MUC7*∗5 allele, it will lack some glycosylation sites indicating differences in the bacterial interaction^35^.

About family history of asthma, in our research, a statistically significant difference was seen between asthmatic children and healthy ones (*p* < 0.001). This adds further evidence that a family history of asthma elevates the risk of asthma incidence in children^36^. Martinez et al. added that parental history of asthma is linked most firmly with early-onset asthma that lasts for higher age^37^. This suggests genetically predisposed children are at significant threat of early-onset of persistent asthma and inspires future studies on the interaction between environmental and genetic factors in such populations.

Designation of markers that relate to the clinical manifestations and airway blockage would be a valuable addition to asthma clinical staging. Asthmatics show chronic inflammation and airways remodelling. Thus, biomarkers of inflammation and remodelling might be beneficial in asthma severity assessment. Lately, investigations have determined various crucial molecules connected with asthma phenotypes, as IgE, TNF-α, and TGF-β1^38–40^.

The predisposing element, widely estimated, for the onset of allergic asthma is over IgE production as a response to the surrounded allergens, particularly when sensitization occurs early in childhood^41^. IgE attaches to specific receptors present on the effector cells e.g., mast cells and basophils. Allergen interacts with IgE and starts inflammation-cascade leading to pro-inflammatory mediators’ flow that takes part in the acute and chronic symptoms of asthma^42^. Regardless of the inaccuracy of serum total IgE in the diagnosis of allergic diseases, modern research has established that it can aid in characterizing refractory asthma phenotypes and configuration of add-on therapies, like anti-IgE monoclonal antibodies, for cases suffering from moderate to severe persistent, uncontrolled asthma^43^. There was no evidence in our study into the association between MUC7 and serum IgE levels in asthmatic individuals.

Macrophages are terminally differentiated innate immune cells that take part in tissue remodelling. Macrophages membrane-bound receptors and generated cytokines direct their variable functions^44^‏. TNF-α is a pro-inflammatory cytokine primarily synthesized by macrophages^45^. It is responsible for the stimulation of local inflammation and induction of acute-phase reaction^46^. It principally participates in starting airway inflammation and making the airway hyper-reactive^47^. Its level was increased in asthma cases samples; sputum, Broncho- alveolar lavage, and lung biopsy^48^. Moreover, TNF-α mRNA and protein expression were increased in the airways of asthmatics, indicating that TNF-α plays a role in the airway inflammatory response in asthma^49,50^. In vitro, the TNF-α was seen to stimulate the *MUC7* gene on promoter activity and *MUC7* mRNA levels^51^.

In asthmatic lungs, transforming growth factor-beta 1 (TGF-β1) is a clue mediator implicated in tissue remodeling^40^. TGF-β1 is the main fibro-genic cytokine^52^. It is a fibro-genic and immune-modulatory factor generated by many cells, including epithelial cells, macrophages, eosinophils, and fibroblasts^53^. In in vitro as well as in vivo studies, a dual role has been shown, acting as a pro- or anti-inflammatory cytokine, and participating in initiating and terminating inflammatory and immunologic responses in the airways^54^. Many researchers detected elevations in TGF-β1 expression in asthma patients. It is yet a controversial issue if the TGF-β1 level correlates to asthma severity or not^55^. In asthma, bronchial epithelial cells' secretion of TGF-β2, not TGF-β1, was suggested to up-regulate the expression of airway mucin^56^.

Despite higher serum levels of IgE, TNF-α, and TGF-β1 in the asthmatic children than controls in our study, the *MUC7* VNTR variant was not associated with any of these levels. Similarly, the *MUC7* VNTR variant was not associated with the clinical manifestation of asthma disease. This could be explained by the small sample size and the limited clinical characteristics evaluated.

Points of strength of the current study: to the best of our knowledge, this is the first study, which investigates *MUC7* VNTR variant and its association with TNF-α, and TGF-β1 serum levels in Egyptian children with bronchial asthma.

Limitations of the study: a single-centre study with a small sample size and a small number of clinical variables. To generalize our results, we recommend larger collaborative multi-centre studies.

## Conclusions

In conclusion, the current study results demonstrated that, among Egyptian children, the MUC7*5 allele might have a protective role against bronchial asthma. Our findings also indicated that the *MUC7* VNTR variant was not associated with the clinical manifestations of bronchial asthma nor with serum levels of IgE, TNF-α, and TGF-β1 in Egyptian children affected with bronchial asthma.

## Data Availability

The datasets generated during and/or analyzed during the current study are available from the corresponding author on reasonable request.
